# Gout and allopurinol adherence in newly diagnosed patients and the risk of fractures: a nationwide cohort study

**DOI:** 10.1007/s10067-026-08298-2

**Published:** 2026-07-17

**Authors:** Jang Ho Doo, Jiwon Yu, Sun Jae Park, Sang woo Park, Jina Chung, Ju Hyun Kang, Young Jun Park, Hyun-Young Shin, Changho Han, Byeongzu Ghang, Sang Min Park

**Affiliations:** 1 Department of Biomedical Sciences, Seoul National University College of Medicine, Seoul, South Korea; 2 Department of Family Medicine, Seoul National University Hospital, Seoul, South Korea; 3 Medical Research Center, Genomic Medicine Institute Seoul National University , Seoul, South Korea; 4 Department of Family Medicine, College of Medicine, Seoul St. Mary’s Hospital, The Catholic University of Korea, Seoul, South Korea; 5 Medical Big Data Research Center, Seoul National University Medical Research Center, Seoul National University College of Medicine, Seoul, South Korea; 6 Department of Rheumatology, Uijeongbu Eulji Medical Center, Uijeongbu, South Korea

**Keywords:** Allopurinol, Gout, Medication adherence, Nationwide cohort study, Osteoporotic fractures, Urate-lowering therapy

## Abstract

**Introduction / Objectives:**

The association between gout and fractures remains controversial due to the competing effects of uric acid's antioxidant properties and gout-induced chronic inflammation. Our study aimed to evaluate the risk of fractures in newly diagnosed gout patients and to analyze the impact of Allopurinol medication adherence on this risk.

**Methods:**

This nationwide cohort study utilized the National Health Insurance Service-National Health Screening Cohort (NHIS-HEALS) database (2002–2019). We identified 4,107 patients newly diagnosed with gout who remained on continuous allopurinol therapy during the 24-month medication assessment period and matched them 1:3 with 12,321 non-gout controls using propensity score matching. Allopurinol adherence was assessed via the Medication Possession Ratio (MPR) over a 24-month period and categorized into three groups: MPR < 0.3, 0.3 ≤ MPR < 0.8, and MPR ≥ 0.8. Multivariable Cox proportional hazards regression was used to calculate adjusted hazard ratios (aHR) and 95% confidence intervals (CI) for fractures (vertebral, hip, and distal radius).

**Results:**

Gout patients demonstrated a significantly higher risk of overall fractures compared to the non-gout group (aHR 5.52; 95% CI 4.19–7.26). A significant inverse linear relationship was observed between allopurinol adherence and fracture risk (P for trend < .001). The risk was highest in the low-adherence group (MPR < 0.3; aHR 5.91; 95% CI 4.40–7.93) and relatively lower in the high-adherence group (MPR ≥ 0.8; aHR 4.77; 95% CI 2.94–7.72). Consistent trends were observed for vertebral (aHR 5.68) and hip (aHR 4.22) fractures. These associations remained consistent across all subgroups, including age, sex, and comorbidities.

**Conclusion:**

Newly diagnosed gout is associated with a substantially increased risk of fractures. Higher adherence to Allopurinol therapy is correlated with a significant reduction in this risk, suggesting that consistent urate-lowering therapy may mitigate gout-related bone fragility.

**Table:** 

**Key Points** *• Newly diagnosed gout patients have a significantly higher risk of major osteoporotic fractures compared to the non-gout population.* *• A significant inverse linear relationship exists between Allopurinol adherence and fracture risk, with the highest adherence group (MPR ≥ 0.8) showing a relatively lower risk.* *• Consistent urate-lowering therapy (ULT) may mitigate bone fragility in gout patients by reducing systemic inflammatory burden caused by urate crystal deposition.*

**Supplementary Information:**

The online version contains supplementary material available at https://doi.org/10.1007/s10067-026-08298-2.

## Introduction

Gout is characterized by recurrent episodes of acute monoarthritis resulting from the intra-articular deposition of monosodium urate crystals. While initially presenting as intermittent flares, the disease may progress to chronic arthritis distinguished by the formation of tophi. Following the resolution of an acute episode, urate-lowering therapy (ULT) is typically indicated for patients presenting with two or more annual flares, progressive joint involvement, or associated comorbidities [1]. Global gout incidence and prevalence have increased steadily over the last 20 years, with the most significant burden observed in high-sociodemographic index regions [2]. The burden of gout in Korea has intensified over the last decade, with a significant rise in incidence and prevalence observed between 2007 and 2015 [3].

The relationship between gout, serum uric acid (SUA) levels, and the risk of bone fractures remains a subject of significant academic debate, with existing literature offering conflicting evidence. On one hand, several studies suggest that gout acts as a risk factor for bone loss; for instance, some population-based research indicates a modest increase in the incidence of osteoporosis and fractures, particularly in women and specific anatomical sites such as the spine and limbs [4, 5]. Conversely, other large-scale meta-analyses and cohort studies have failed to find a statistically significant association between gout and increased fracture risk [6, 7].

Furthermore, the biological role of uric acid itself is controversial. While some epidemiological and experimental evidence suggests that uric acid may exert a protective antioxidant effect on bone metabolism, thereby increasing bone mineral density and reducing fracture risk in certain populations [8, 9], other longitudinal data indicate that elevated SUA levels may actually correlate with a higher risk of hip fractures [10]. Similarly, the impact of ULT remains unclear, as studies vary on whether achieving target uric acid levels provides a tangible benefit in reducing incident fractures [11] or has no significant effect on long-term outcomes [12].

Despite extensive research, it remains unclear whether gout independently increases fracture risk compared to non-gout populations, and the extent to which ULT modulates this relationship remains poorly defined. Given that medication adherence among gout patients is notoriously poor [13, 14], it is essential to investigate how fracture risk in gout patients deviates from the general population and the degree to which adherence levels modulate this risk. Therefore, this study utilizes a large-scale, population-based cohort to evaluate the association between gout and fractures, with a specific focus on the impact of medication adherence as measured by the medication possession ratio (MPR).

## Materials and methods

### Data sources

This study utilized the National Health Insurance Service-National Health Screening Cohort (NHIS-HEALS), covering the period from 2002 to 2019. This database is a representative sample of individuals who underwent health screenings in 2002–2003 and has been utilized extensively in prior medical research. Because South Korea employs a universal healthcare system, the NHIS serves as the sole provider, offering comprehensive longitudinal data. The dataset includes biennial health screening results, such as physical measurements, lab work, and lifestyle surveys, alongside detailed clinical records regarding diagnoses, medications, and procedures. Additionally, it contains demographic variables, such as, income, age, and geographic location [15].

### Study population

**Fig. 1:**
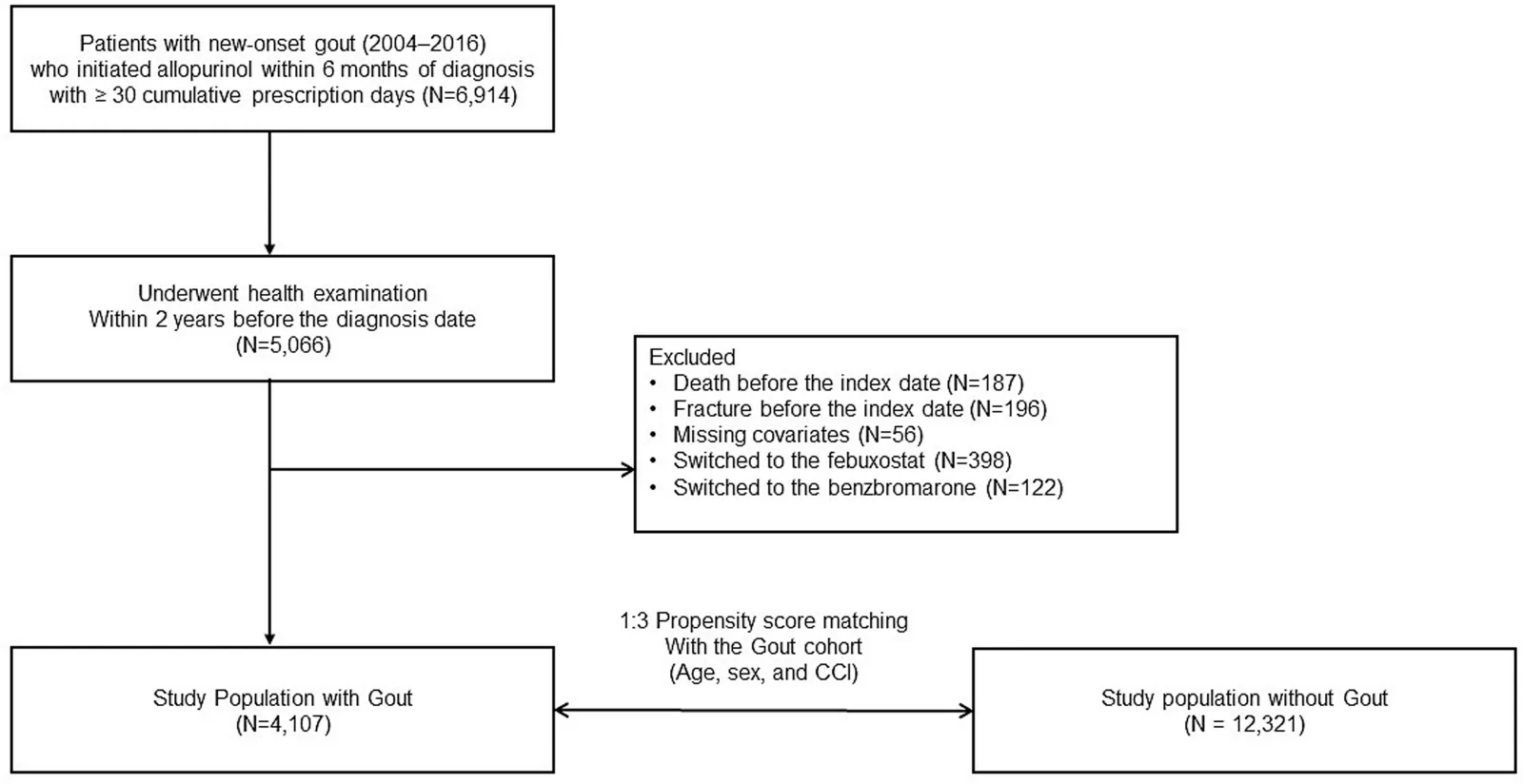
Note: This data is mandatory. Please provide

Initially, 6,914 individuals were identified between 2004 and 2016 who received a diagnosis of gout for the first time, according to the International Statistical Classification of Diseases and Related Health Problems, 10th Revision (ICD-10) code M10, with a concurrent allopurinol prescription initiated within six months of the diagnosis. To focus on patients requiring consistent urate-lowering therapy, inclusion was restricted to those with at least 30 cumulative days of allopurinol. To ensure the availability of baseline health data (lifestyle, lab results, and anthropometrics), we further selected 5,066 patients who had undergone a health screening within two years prior to diagnosis. Because the Medication Possession Ratio (MPR) was calculated over a two-year period following the start of treatment, the index date was defined as two years post-diagnosis. The 24-month period between the initial gout diagnosis and the index date was used exclusively to assess medication adherence. Follow-up for fracture outcomes began from the index date and continued until fracture occurrence, death, or December 31, 2019, whichever came first. Patients were excluded if they died (n = 187) or suffered a fracture (n = 196) before this index date, or if they had missing covariate data (n = 56). During the initial 24-month medication assessment period, 398 patients who switched from allopurinol to febuxostat and 122 patients who switched from allopurinol to probenecid were excluded from the analysis to minimize potential bias related to treatment switching. Consequently, the final study population comprised 4,107 patients who remained on continuous allopurinol therapy throughout the medication assessment period. To provide a comparative analysis, a control group without gout was established from the NHIS-HEALS database through 1:3 propensity score matching (PSM). These individuals were matched to the gout cohort (N = 4,107) based on age, sex, and Charlson Comorbidity Index (CCI), resulting in a non-gout study population of 12,321 participants. We defined the index date as two years post-diagnosis (or two years after the assigned reference date of June 30th for controls) to accommodate a comprehensive 24-month assessment of allopurinol MPR. All baseline clinical characteristics, including CCI and health examination data, were evaluated during the two-year period preceding the initial diagnosis or reference date. The CCI was calculated using the ICD-10 coding algorithms validated in previous literature [16]. (Fig. 1).

### Key variables

The medication possession ratio (MPR) for allopurinol was calculated by dividing the total number of prescribed days by the 24-month observation period. The period included in the analysis was between the diagnosis date and two years after the diagnosis date. The MPR was capped at 100% since it was not plausible for the patients to take allopurinol more times than prescribed. The gout study population was divided into three categories, MPR < 0.3, 0.3 ≤ MPR < 0.8, MPR ≥ 0.8.

The primary outcome was the incidence of fractures, which were classified into three distinct categories. Overall fracture was defined as a composite of vertebral (ICD-10: S22.0, S22.1, S32.0, M48.4, M48.5), hip (S72.0, S72.1), and distal radius (S52.5, S52.6) fractures. To ensure diagnostic accuracy, we only included events requiring hospitalization for at least two days, a rigorous definition validated in previous literature. [17, 18] We evaluated the risk of overall, vertebral, hip, and other fractures in the gout cohort relative to the non-gout control group, further stratifying the results by Allopurinol MPR levels.

Covariates included age (continuous), sex (categorical), CCI (continuous), and household income (categorical; 1 st [lowest], 2nd, 3rd, and 4th [highest]). Lifestyle factors included smoking status (categorical; never, former, and current), alcohol intake (categorical; none, 1–2, 3–4, and ≥ 5 times/week), and physical activity (categorical; none, 1–2, 3–4, and ≥ 5 times/week). Additionally, body mass index (BMI, continuous, kg/m^2^), systolic blood pressure (continuous, mmHg), total cholesterol (continuous, mg/dL), and fasting blood glucose (continuous, mg/dL) were extracted from the health examination data during the two years prior to the gout diagnosis or reference date. Hypertension was defined as having ICD-10 codes of I10–I13 or I15 and a prescription for antihypertensive drugs. Dyslipidemia was defined as having code E78 and lipid-lowering drugs. Diabetes mellitus was defined as having codes E11–E14 and a prescription record of glucose-lowering drugs. CKD was considered present when a participant had at least one hospitalization or three outpatient visits with a CKD diagnosis code, consistent with previous NHIS studies [19]. The presence of these four diseases was checked between the diagnosis or reference date and two years before that date. Previous systemic corticosteroid exposure was identified using prescription claims before the index date and was included in the baseline characteristics. Participants were followed from the index date until the occurrence of a fracture event, death, or December 31, 2019, whichever came first.

### Statistical analysis

Multivariable Cox proportional hazard regression models were used to assess the risk of fracture according to the presence of gout compared to non-gout patients, and according to the allopurinol MPR. Model 1 was adjusted for age and sex. Model 2 was further adjusted for household income, BMI, CCI, and underlying comorbidities (hypertension, dyslipidemia, and diabetes mellitus). Model 3 (the fully adjusted model) included all variables in Model 2 plus lifestyle factors, such as smoking status, alcohol consumption, and physical activity. P for trend was calculated across four groups for each outcome: non-gout (reference), MPR ≥ 0.8, 0.3 ≤ MPR < 0.8, and MPR < 0.3.

Subgroup analyses were performed based on age (< 60 vs. ≥ 60 years), sex (male vs. female), household income (1st–2nd vs. 3rd–4th quartiles), smoking status (never vs. former/current), physical activity (no vs. yes), systemic corticosteroid use (no vs. yes), CCI (0 vs. ≥ 1), and BMI (< 25 vs. ≥ 25 kg/m^2^). All subgroups were analyzed using Model 3, adjusting for all covariates except for the specific variable used as the stratification factor. The P for interaction was calculated to assess whether the association between gout/MPR status and fracture risk was modified by each stratification variable. All analyses were conducted using SAS software version 9.4 (SAS Institute Inc., Cary, NC, USA). Statistical significance was defined as a two-sided p-value of less than 0.05, and a standardized mean difference (SMD) of less than 0.1 was considered to indicate no significant differences between groups. Continuous variables were expressed as mean (standard deviation), while categorical variables were presented as percentages. As a sensitivity analysis, the risk of overall fracture was additionally evaluated separately within the first and second years of follow-up (Supplementary Table S2).

## Results

Table 1 presents the baseline characteristics of the study population, comprising 4,107 gout patients and 12,321 matched controls. Through 1:3 propensity score matching, age (mean 60.29 years), sex (91.3% male), and CCI were perfectly balanced (Standardized Mean Difference = 0.000). However, several variables exceeded the SMD threshold of 0.1, indicating remaining imbalances. Compared to controls, patients with gout had a higher BMI (25.24 vs. 23.89 kg/m^2^, SMD = 0.473), systolic blood pressure (130.71 vs. 126.99 mmHg, SMD = 0.241), and total cholesterol (199.97 vs. 195.34 mg/dL, SMD = 0.119), along with a substantially higher prevalence of hypertension (60.2% vs. 32.9%, SMD = 0.571), dyslipidemia (32.5% vs. 27.0%, SMD = 0.120), diabetes mellitus (17.6% vs. 9.9%, SMD = 0.225), chronic kidney disease (3.5% vs. 0.1%, SMD = 0.256), and previous systemic corticosteroid use (43.7% vs. 26.2%, SMD = 0.372). Regarding lifestyle factors, the gout group had a higher proportion of never smokers (49.7% vs. 42.6%, SMD = 0.155) and reported lower levels of physical activity (SMD = 0.154). Among 4,107 gout patients, baseline characteristics significantly differed according to medication adherence levels (Supplementary Table S1).

**Table 1:** Baseline characteristics of the study population according to gout status and medication adherence (MPR)

Variable	Non-gout	Gout	Standardized Mean Difference (SMD)
No. of participants, n (%)	12,321	4,107	
Age, years, mean (SD)	60.29 (9.27)	60.29 (9.27)	0.000
Age group, n (%)			
40–49	1,482 (12.0)	494 (12.0)	0.051
50–59	4,675 (37.9)	1,625 (39.6)	
60–69	3,891 (31.6)	1,197 (29.1)	
≥ 70	2,373 (19.3)	791 (19.3)	
Sex, n (%)			
Male	11,250 (91.3)	3,750 (91.3)	0.000
Female	1,071 (8.7)	357 (8.7)	
Charlson Comorbidity Index, n (%)			
0	3,495 (28.4)	1,165 (28.4)	0.000
1	3,342 (27.1)	1,114 (27.1)	
≥ 2	5,484 (44.5)	1,828 (44.5)	
Household income, n (%)			
1st	2,465 (20.0)	691 (16.8)	0.099
2nd	2,705 (22.0)	875 (21.3)	
3rd	2,567 (20.8)	867 (21.1)	
4th	4,584 (37.2)	1,694 (41.2)	
Smoking status, n (%)			
Never	5,250 (42.6)	2,041 (49.7)	0.155
Former	4,070 (33.0)	1,103 (26.9)	
Current	3,001 (24.4)	963 (23.4)	
Alcohol intake (times/week), n (%)			
None	5,712 (46.4)	1,972 (48.0)	0.089
1–2	4,112 (33.4)	1,210 (29.5)	
3–4	1,583 (12.8)	594 (14.5)	
≥ 5	914 (7.4)	331 (8.1)	
Physical activity (times/week), n (%)			
None	5,150 (41.8)	1,755 (42.7)	0.154
1–2	2,146 (17.4)	913 (22.2)	
3–4	1,838 (14.9)	588 (14.3)	
≥ 5	3,187 (25.9)	851 (20.7)	
Body mass index, kg/m^2^, mean (SD)	23.89 (2.83)	25.24 (2.88)	0.473
Systolic blood pressure, mmHg, mean (SD)	126.99 (14.92)	130.71 (15.98)	0.241
Total cholesterol, mg/dL, mean (SD)	195.34 (36.63)	199.97 (41.09)	0.119
Fasting blood glucose, mg/dL, mean (SD)	102.01 (21.87)	102.07 (24.25)	0.003
Hypertension			
No	8,271 (67.1)	1,633 (39.8)	0.571
Yes	4,050 (32.9)	2,474 (60.2)	
Dyslipidemia			
No	8,990 (73.0)	2,771 (67.5)	0.120
Yes	3,331 (27.0)	1,336 (32.5)	
Diabetes mellitus			
No	11,097 (90.1)	3,383 (82.4)	0.225
Yes	1,224 (9.9)	724 (17.6)	
Chronic Kidney Disease			
No	12,305 (99.9)	3,962 (96.5)	0.256
Yes	16 (0.1)	145 (3.5)	
Systemic corticosteroid use			
No	9,092 (73.8)	2,314 (56.3)	0.372
Yes	3,229 (26.2)	1,793 (43.7)	

Data expression: Continuous variables are expressed as mean (SD), and categorical variables are expressed as number (percentage)

Standardized Mean Difference (SMD): An SMD > 0.1 is considered to indicate a significant clinical difference in baseline characteristics between groups

Household income: Categorized into quartiles (1st, lowest; 4th, highest) based on health insurance premium levels

Physical activity: Defined as the weekly frequency of physical activity (times per week)

Charlson Comorbidity Index (CCI): A method of categorizing comorbidities based on ICD diagnosis codes

*SD*, standard deviation; *SMD*, standardized mean difference;* BMI*, body mass index; *SBP*, systolic blood pressure; *CCI*, Charlson comorbidity index

Table 2 presents the risk for major osteoporotic fractures according to gout status and allopurinol medication adherence. During the follow-up period, the incidence rate for overall fracture was higher in the gout group (5.274 per 1,000 person-years) than in the non-gout group (1.286 per 1,000 person-years). In the fully adjusted model (Model 3), the gout group exhibited a significantly increased risk of overall fracture (aHR 5.52; 95% CI 4.19–7.26). When stratified by medication adherence, a significant dose–response relationship was observed (P for trend < 0.001), with the highest risk found in the low-adherence group (MPR < 0.3; aHR 5.91; 95% CI 4.40–7.93) and a relatively lower risk in the high-adherence group (MPR ≥ 0.8; aHR 4.77; 95% CI 2.94–7.72). This trend was consistently observed across vertebral, hip, and other fractures. For vertebral fractures, gout patients had an increased risk (aHR 5.68; 95% CI 3.98–8.11), with the risk inversely associated with medication adherence (P for trend < 0.001; MPR < 0.3 aHR 6.60 vs. MPR ≥ 0.8 aHR 4.37). The risk for hip fractures was also elevated in the gout cohort (aHR 4.22; 95% CI 2.36–7.57), showing a similar significant trend (P for trend < 0.001). For other fractures, gout was associated with a higher risk (aHR 6.25; 95% CI 3.34–11.68), which remained significant across all MPR levels (P for trend < 0.001). Additional sensitivity analyses according to follow-up duration showed that the increased risk of overall fracture remained significant during both the first and second years of follow-up (Supplementary Table S2).

**Table 2:** Risk of fractures according to gout status and medication adherence (MPR) after propensity score matching

Outcome	Non-Gout	Gout Overall	Gout by Medication Adherence (MPR)			P for trend
			MPR ≥ 0.8	0.3 ≤ MPR < 0.8	MPR < 0.3	
Overall Fracture						
Events, n	94	155	22	30	103	
Person-years	73,085	29,387	4,343	6,349	18,604	
Incidence rate*	1.286	5.274	5.065	4.725	5.536	
aHR (95% CI)						
Model 1ᵃ	1 (Reference)	4.46 (3.45–5.78)	3.69 (2.32–5.87)	3.88 (2.57–5.87)	4.91 (3.70–6.51)	< 0.001
Model 2ᵇ	1 (Reference)	5.43 (4.14–7.13)	4.64 (2.87–7.50)	4.77 (3.12–7.29)	5.85 (4.37–7.83)	< 0.001
Model 3ᶜ	1 (Reference)	5.52 (4.19–7.26)	4.77 (2.94–7.72)	4.88 (3.19–7.48)	5.91 (4.40–7.93)	< 0.001
Vertebral fracture						
Events, n	55	94	12	15	67	
Person-years	73,111	29,654	4,374	6,408	18,751	
Incidence rate*	0.752	3.170	2.743	2.340	3.573	
aHR (95% CI)						
Model 1ᵃ	1 (Reference)	4.70 (3.36–6.58)	3.41 (1.83–6.38)	3.29 (1.85–5.83)	5.64 (3.94–8.09)	< 0.001
Model 2ᵇ	1 (Reference)	5.65 (3.97–8.05)	4.28 (2.25–8.17)	3.92 (2.18–7.06)	6.59 (4.54–9.58)	< 0.001
Model 3ᶜ	1 (Reference)	5.68 (3.98–8.11)	4.37 (2.29–8.35)	3.98 (2.22–7.18)	6.60 (4.53–9.62)	< 0.001
Hip fracture						
Events, n	24	29	5	8	16	
Person-years	73,135	29,724	4,399	6,414	18,910	
Incidence rate*	0.328	0.976	1.136	1.247	0.846	
aHR (95% CI)						
Model 1ᵃ	1 (Reference)	3.14 (1.82–5.42)	3.13 (1.19–8.21)	3.92 (1.76–8.77)	2.86 (1.51–5.41)	< 0.001
Model 2ᵇ	1 (Reference)	3.96 (2.23–7.06)	4.17 (1.53–11.40)	4.98 (2.15–11.51)	3.58 (1.85–6.93)	< 0.001
Model 3ᶜ	1 (Reference)	4.22 (2.36–7.57)	4.34 (1.58–11.95)	5.36 (2.30–12.50)	3.83 (1.98–7.45)	< 0.001
Other fracture						
Events, n	16	34	5	8	21	
Person-years	73,085	29,297	4,343	6,349	18,604	
Incidence rate*	0.219	1.161	1.151	1.260	1.129	
aHR (95% CI)						
Model 1ᵃ	1 (Reference)	5.42 (2.97–9.87)	5.21 (1.91–14.26)	6.11 (2.60–14.35)	5.24 (2.72–10.11)	< 0.001
Model 2ᵇ	1 (Reference)	6.26 (3.37–11.63)	5.94 (2.11–16.77)	7.08 (2.95–17.03)	6.08 (3.11–11.89)	< 0.001
Model 3ᶜ	1 (Reference)	6.25 (3.34–11.68)	6.11 (2.15–17.35)	7.16 (2.96–17.30)	6.01 (3.05–11.82)	< 0.001

P for trend was calculated across four groups: Non-gout (reference), Gout with MPR ≥ 0.8, 0.3 ≤ MPR < 0.8, and MPR < 0.3

*MPR*, medication possession ratio; *aHR*, adjusted hazard ratio; *CI*, confidence interval; *IR*, incidence rate; *PY*, person-years

*Incidence rate: Expressed per 1,000 person-years

^a^Model 1: Adjusted for age and sex

^b^Model 2: Adjusted for Model 1 variables plus income Household Income, Body Mass Index (BMI), Charson Comorbidity Index (CCI), and Underlying Comorbidities (hypertension, dyslipidemia, and diabetes mellitus)

^c^Model 3: Adjusted for Model 2 variables plus lifestyle factors including smoking status, alcohol consumption, and physical activity

Subgroup analysis demonstrated that the increased risk of major osteoporotic fractures associated with gout and low medication adherence was consistent across all analyzed strata (Table 3). The risk of fracture remained significantly elevated in the gout cohort regardless of age, sex, household income, alcohol intake, smoking status, physical activity, systemic corticosteroid use, CCI, or BMI (all P for trend < 0.001). No statistically significant interactions were observed between gout/MPR status and any stratification variables, including BMI (P for interaction = 0.338), physical activity (P for interaction = 0.862), and systemic corticosteroid use (P for interaction = 0.148). These findings indicate that the association between gout, allopurinol adherence, and fracture risk was generally consistent across the analyzed subgroups.

**Table 3:** Subgroup Analysis of fracture risks According to Gout Status and Medication Adherence (MPR)

Subgroup	Non-Gout	Gout Overall	Gout			P for interaction	P for trend
			MPR ≥ 0.8	0.3 ≤ MPR < 0.8	MPR < 0.3		
Age							
< 60 years	1 (Reference)	8.09 (4.25–15.44)	11.55 (4.22–31.63)	9.56 (3.85–23.77)	7.24 (3.63–14.43)	0.141	< 0.001
≥ 60 years	1 (Reference)	4.62 (3.41–6.27)	3.45 (1.98–6.01)	3.82 (2.35–6.23)	5.25 (3.78–7.29)		< 0.001
Sex							
Male	1 (Reference)	5.64 (4.09–7.79)	5.26 (3.05–9.08)	5.26 (3.05–9.08)	5.91 (4.17–8.39)	0.851	< 0.001
Female	1 (Reference)	5.60 (3.31–9.50)	3.53 (1.19–10.47)	4.58 (1.93–10.85)	6.48 (3.68–11.42)		< 0.001
Household income							
1 st, 2nd quartile	1 (Reference)	5.77 (3.89–8.52)	3.99 (1.89–8.15)	5.05 (2.71–9.40)	6.48 (4.27–9.84)	0.543	< 0.001
3rd, 4th quartile	1 (Reference)	5.42 (3.68–7.98)	5.61 (2.95–10.65)	5.13 (2.85–9.26)	5.46 (3.58–8.31)		< 0.001
Alcohol intake							
No	1 (Reference)	5.89 (4.20–8.31)	5.79 (3.31–10.14)	4.29 (2.43–7.57)	6.47 (4.49–9.34)	0.787	< 0.001
Yes	1 (Reference)	5.01 (3.15–*7.98*)	2.94 (1.11–7.79)	5.89 (3.03–11.35)	5.20 (3.16–8.56)		< 0.001
Smoking status							
Never	1 (Reference)	4.99 (3.49–7.15)	3.99 (2.09–7.42)	3.68 (2.02–6.54)	5.75 (3.93–8.42)	0.443	< 0.001
Former/Current	1 (Reference)	6.68 (4.40–10.24)	6.67 (3.19–13.96)	7.43 (3.97–13.91)	6.45 (4.03–10.32)		< 0.001
Physical activity							
No	1 (Reference)	5.37 (3.78–7.64)	5.09 (2.71–9.57)	5.11 (2.99–8.73)	5.52 (3.77–8.08)	0.862	< 0.001
Yes	1 (Reference)	5.71 (3.69–8.84)	4.33 (2.04–9.18)	4.60 (2.28–9.29)	6.52 (4.08–10.42)		< 0.001
Systemic corticosteroid use							
No	1 (Reference)	4.97 (3.49–7.08)	4.66 (2.50–8.67)	4.22 (2.39–7.45)	5.32 (3.62–7.82)	0.148	< 0.001
Yes	1 (Reference)	6.25 (3.92–9.97)	5.02 (2.30–10.96)	5.93 (3.04–11.57)	6.64 (4.06–10.87)		< 0.001
Charlson comorbidity index (CCI)							
0	1 (Reference)	5.66 (2.79–11.45)	2.95 (0.63–13.94)	7.64 (3.12–18.66)	5.38 (2.54–11.43)	0.375	< 0.001
≥ 1	1 (Reference)	5.49 (4.07–7.42)	5.18 (3.11–8.62)	4.18 (2.52–6.93)	5.99 (4.34–8.27)		< 0.001
Body Mass Index (BMI)							
< 25 kg/m^2^	1 (Reference)	5.55 (3.99–7.72)	3.99 (1.98–7.67)	5.68 (3.39–9.51)	5.94 (4.14–8.53)	0.338	< 0.001
≥ 25 kg/m^2^	1 (Reference)	4.16 (2.62–6.59)	4.30 (2.14–8.64)	2.99 (1.38–6.07)	4.57 (2.79–7.50)		< 0.001

Values are presented as Hazard Ratio (95% CI) unless otherwise indicated

All subgroups were analyzed using the fully adjusted model (Model 3), which includes age, sex, income, BMI, CCI, hypertension, dyslipidemia, diabetes mellitus, smoking status, alcohol consumption, and physical activity, except for the variable used as the stratification factor

P for interaction represents the p-value for the interaction between the gout/MPR status and each stratification variable (e.g., Age < 60 vs. ≥ 60)

P for trend was calculated across four groups: Non-gout (reference), Gout with MPR ≥ 0.8, 0.3 ≤ MPR < 0.8, and MPR < 0.3

*MPR*, medication possession ratio; *aHR*, adjusted hazard ratio; *CI*, confidence interval; *CCI*, Charlson comorbidity index; *BMI*, body mass index

## Discussion

This nationwide cohort study demonstrated that patients with gout had a higher risk of fractures than matched non-gout controls. In addition, fracture risk tended to be higher among patients with lower adherence to allopurinol therapy, suggesting that consistent allopurinol use may be associated with a relatively lower fracture risk. These relationships remained consistent even after performing subgroup analysis, which indicates that medication adherence does not differently affect the risk of fractures among different groups.

The finding that gout is associated with an increased risk of fractures (aHR 5.52) is consistent with several population-based studies [4, 20]. For instance, Tzeng et al. reported a higher fracture risk (aHR 1.17) in a Taiwanese gout cohort [4], and Paik et al. observed a modest increase in hip fracture risk among women with gout [5]. Conversely, these results differ from certain meta-analyses and cohorts that reported no significant association [3, 6, 7]. While some epidemiological evidence suggests a potential bone-protective effect of serum uric acid due to its antioxidant properties [8, 9], the current data indicate that in this study population, gout status remains a risk factor for fracture.

Regarding urate-lowering therapy, the observed inverse linear relationship between allopurinol adherence and fracture risk (P for trend < 0.001) aligns with the results of Wei et al., who found that achieving target serum urate levels significantly lowered fracture risk (HR 0.66) [11]. This contrasts with earlier studies that found no significant reduction in fracture risk with allopurinol use [6, 7, 12]. The discrepancy may be attributed to the categorization of medication adherence; while some studies evaluated allopurinol use as a binary variable or by duration [12], the present study utilized MPR categories, demonstrating that the association with reduced risk is specifically prominent in the high-adherence group (MPR ≥ 0.8).

The results show that only 16.0% of patients maintained high allopurinol adherence (MPR ≥ 0.8), a rate lower than the 23.2% to 42% reported in previous studies [12, 14, 21, 22]. Consistent with prior data, patients in the high adherence group were older and exhibited a higher burden of comorbidities at baseline. Despite these clinical profiles, the inverse relationship between medication adherence and fracture risk remained significant. These findings suggest that consistent allopurinol use may be associated with lower fracture risk regardless of the higher baseline vulnerability in the high-adherence cohort, highlighting the potential role of continuous urate-lowering therapy in fracture risk management.

The relationship between uric acid (UA) and bone health is characterized by a dual nature: while physiological levels may provide antioxidant-driven bone protection [8, 9, 23], our findings suggest that in gout patients, this potential benefit is significantly outweighed by a dominant pro-inflammatory effect driven by systemic urate deposition. Beyond its role as a circulating antioxidant, pathological hyperuricemia leads to the formation of monosodium urate (MSU) crystals which, as recent evidence underscores, are not confined to peripheral joints but are deposited extensively in extra-articular tissues, including the spine, vasculature, and various internal organs [24]. These systemic deposits function as chronic inflammatory nidi; the presence of MSU crystals triggers a robust inflammatory cascade characterized by the infiltration of lymphocytes and macrophages [25, 26]. This persistent inflammatory milieu elevates key mediators such as IL-1 and RANKL, which directly promote osteoclastogenesis and accelerate bone resorption [24–26]. Consequently, the observed inverse relationship between allopurinol adherence and fracture risk may suggest that consistent urate-lowering therapy reduces the net inflammatory burden on the skeleton by facilitating the dissolution of systemic crystal deposits. In addition, previous experimental evidence suggests that uric acid may influence bone metabolism through the vitamin D–PTH axis; however, this mechanism could not be directly evaluated in the present study because serum urate, vitamin D, and PTH levels were unavailable [27].

This study has several strengths. First, the use of a large-scale, nationwide cohort provided sufficient statistical power to account for a wide range of covariates, including comorbidities and lifestyle behaviors. Second, by categorizing gout patients based on the MPR, this study identified a significant inverse association between allopurinol adherence and fracture risk. Furthermore, the 1:3 propensity score matching with a non-gout control group allowed for a robust comparison of fracture incidence associated with gout status. However, certain limitations should be considered. First, laboratory data on serum uric acid (sUA) levels were not available in the NHIS-HEALS database. Although medication adherence is strongly correlated with achieving target sUA levels [13], we could not directly confirm whether high MPR led to biochemical control of hyperuricemia. Second, the severity of gouty arthritis, such as the frequency of acute flares or the presence of tophi, could not be individually identified. Third, because MPR was calculated by prescription records, we could not definitively identify whether patients actually ingested the prescribed medications. In addition, because the NHIS database does not include synovial fluid crystal identification, imaging findings, or detailed clinical manifestations, gout could not be defined using the 2015 ACR/EULAR classification criteria and was instead identified using an operational definition based on claims data.

In summary, this study demonstrates that gout significantly increases the risk of major osteoporotic fractures. A trend toward lower fracture risk was observed among patients with higher allopurinol adherence, suggesting that consistent long-term allopurinol therapy may be relevant to fracture risk management in gout patients. These findings highlight that consistent, long-term medication adherence may be essential to reducing the possible fracture burden in gout patients.

## Supplementary Information

Below is the link to the electronic supplementary material.

Supplementary file 1 (DOCX 23.6 KB)
