# Supplementary material for: Gout and allopurinol adherence in newly diagnosed patients and the risk of fractures: a nationwide cohort study
Source: Clin Rheumatol. 2026 Jul 17;45(9):5749–58. doi: 10.1007/s10067-026-08298-2 (PMC13526098; doi:10.1007/s10067-026-08298-2)
Supplement: Supplementary file 1 [file 10067_2026_8298_MOESM1_ESM.docx]

Supplementary Table S1. Baseline characteristics of the study population according to gout medication adherence (MPR)

| Variable | Gout | | | P-value |
| --- | --- | --- | --- | --- |
|  | 0.8 ≤ MPR | 0.3 ≤ MPR < 0.8 | MPR < 0.3 |  |
| No. of participants, n (%) | 658 | 869 | 2,580 |  |
| Age, years, mean (SD) | 61.95 (9.20) | 60.63 (9.24) | 59.71 (9.25) | <0.0001 |
| Age group, n (%) |  |  |  | <0.0001 |
| 40–49 | 57 (8.7) | 98 (11.3) | 339 (13.1) |  |
| 50–59 | 233 (35.4) | 326 (37.5) | 1,066 (41.3) |  |
| 60–69 | 211 (32.1) | 271 (31.2) | 715 (27.7) |  |
| ≥70 | 157 (23.9) | 174 (20.0) | 460 (17.8) |  |
| Sex, n (%) |  |  |  | 0.2341 |
| Male | 599 (91.0) | 806 (92.8) | 2,345 (90.9) |  |
| Female | 59 (9.0) | 63 (7.2) | 235 (9.1) |  |
| Household income, n (%) |  |  |  | 0.8689 |
| 1st | 100 (15.2) | 149 (17.1) | 442 (17.1) |  |
| 2nd | 138 (21.0) | 190 (21.9) | 527 (20.4) |  |
| 3rd | 142 (21.6) | 176 (20.3) | 549 (21.3) |  |
| 4th | 278 (42.2) | 354 (40.7) | 1,062 (41.2) |  |
| Smoking Status, n (%) |  |  |  | 0.0792 |
| Never | 332 (50.5) | 433 (49.8) | 1,276 (49.5) |  |
| Former | 189 (28.7) | 249 (28.7) | 665 (25.8) |  |
| Current | 137 (20.8) | 187 (21.5) | 639 (24.8) |  |
| Alcohol intake (times/week) |  |  |  | 0.4448 |
| None | 322 (48.9) | 431 (49.6) | 1,219 (47.2) |  |
| 1–2 | 203 (30.9) | 256 (29.5) | 751 (29.1) |  |
| 0.3–4 | 86 (13.1) | 113 (13.0) | 395 (15.3) |  |
| ≥5 | 47 (7.1) | 69 (7.9) | 215 (8.3) |  |
| Physical activity, times/week, n (%) |  |  |  | 0.3833 |
| None | 258 (39.2) | 336 (38.7) | 1,131 (43.8) |  |
| 1–2 | 154 (23.4) | 198 (22.8) | 561 (21.7) |  |
| 3–4 | 105 (16.0) | 131 (15.1) | 352 (13.6) |  |
| ≥5 | 141 (21.4) | 186 (21.4) | 536 (20.8) |  |
| Body mass index, kg/m², mean (SD) | 25.49 (2.84) | 25.35 (2.87) | 25.14 (2.86) | 0.0102 |
| Systolic blood pressure, mmHg, mean (SD) | 130.31 (16.39) | 131.41 (16.24) | 130.57 (15.80) | 0.3252 |
| Total cholesterol, mg/dL, mean (SD) | 192.76 (43.17) | 200.23 (39.94) | 201.70 (40.74) | <0.0001 |
| Fasting blood glucose, mg/dL, mean (SD) | 104.09 (28.81) | 101.28 (24.78) | 101.85 (22.74) | 0.0569 |
| Charlson Comorbidity Index, n (%) |  |  |  | <0.0001 |
| 0 | 140 (21.3) | 261 (30.0) | 764 (29.6) |  |
| <1 | 150 (22.8) | 211 (24.3) | 753 (29.2) |  |
| ≥2 | 368 (55.9) | 397 (45.7) | 1,063 (41.2) |  |
| Hypertension |  |  |  | <0.0001 |
| No | 121 (18.4) | 307 (35.3) | 1,205 (46.7) |  |
| Yes | 537 (81.6) | 562 (64.7) | 1,375 (53.3) |  |
| Dyslipidemia |  |  |  | <0.0001 |
| No | 338 (51.4) | 589 (67.8) | 1,844 (71.5) |  |
| Yes | 320 (48.6) | 280 (32.2) | 736 (28.5) |  |
| Diabetes Mellitus |  |  |  | <0.0001 |
| No | 480 (72.9) | 697 (80.2) | 2,206 (85.5) |  |
| Yes | 178 (27.1) | 172 (19.8) | 374 (14.5) |  |
| Chronic Kidney Diseaes |  |  |  | <0.0001 |
| No | 605 (91.9) | 826 (95.1) | 2,531 (98.1) |  |
| Yes | 53 (8.1) | 43 (4.9) | 49 (1.9) |  |
| Systemic corticosteroid use |  |  |  | 0.0203 |
| No | 401 (60.9) | 469 (54.0) | 1,444 (56.0) |  |
| Yes | 257 (39.1) | 400 (46.0) | 1,136 (44.0) |  |

Abbreviations: MPR, Medication Possession Ratio; SD, standard deviation.
P values were calculated using χ² tests for categorical variables and one-way ANOVA for continuous variables.

Supplementary Table S2. Risk of overall fractures within 1 year and 2 years according to gout status and medication adherence (MPR)

| Outcome | Non-Gout | Gout Overall | Gout by Medication Adherence (MPR) | | | P for  trend |
| --- | --- | --- | --- | --- | --- | --- |
|  |  |  | MPR ≥ 0.8 | 0.3 ≤ MPR < 0.8 | MPR < 0.3 |  |
| Overall Fracture  within 1 year |  |  |  |  |  |  |
| Events, n | 3 | 16 | 2 | 3 | 11 |  |
| Person-years | 12,303 | 4,074 | 654 | 862 | 2,557 |  |
| Incidence rate* | 0.244 | 3.927 | 3.055 | 3.479 | 4.300 |  |
| Model 1ᵃ | 1 (Reference) | 16.20 (4.72–55.60) | 10.94 (1.83–65.51) | 14.13 (2.85–70.01) | 18.57 (5.18–66.58) |  |
| Model 2ᵇ | 1 (Reference) | 15.25 (4.31–54.02) | 9.40 (1.50–58.80) | 13.07 (2.56–66.70) | 17.65 (4.82–64.63) |  |
| Model 3ᶜ | 1 (Reference) | 15.51 (4.37–55.11) | 9.47 (1.51–59.44) | 13.06 (2.54–67.10) | 18.03 (4.92–66.13) |  |
| Overall Fracture  within 2 years |  |  |  |  |  |  |
| Events, n | 10 | 30 | 5 | 6 | 19 |  |
| Person-years | 23,867 | 7,968 | 1,274 | 1,696 | 4,997 |  |
| Incidence rate* | 0.419 | 3.765 | 3.922 | 3.537 | 3.802 |  |
| aHR (95% CI) |  |  |  |  |  |  |
| Model 1ᵃ | 1 (Reference) | 9.18 (4.49–18.78) | 8.14 (2.78–23.83) | 8.41 (3.06–23.14) | 9.79 (4.55–21.06) |  |
| Model 2ᵇ | 1 (Reference) | 9.79 (4.66–20.58) | 8.65 (2.84–26.38) | 9.13 (3.23–25.77) | 10.30 (4.71–22.65) |  |
| Model 3ᶜ | 1 (Reference) | 9.46 (4.50–19.89) | 8.50 (2.79–25.95) | 8.83 (3.12–25.01) | 9.90 (4.51–21.72) |  |

Abbreviations: MPR, medication possession ratio; aHR, adjusted hazard ratio; CI, confidence interval; IR, incidence rate; PY, person-years.
*Incidence rate: Expressed per 1,000 person-years
^ᵃ^ Model 1: Adjusted for age and sex.
^ᵇ^ Model 2: Adjusted for Model 1 variables plus income Household Income, Body Mass Index (BMI), Charson Comorbidity Index (CCI), and Underlying Comorbidities (hypertension, dyslipidemia, and diabetes mellitus).
^ᶜ^ Model 3: Adjusted for Model 2 variables plus lifestyle factors including smoking status, alcohol consumption, and physical activity.
P for trend was calculated across four groups: Non-gout (reference), Gout with MPR ≥ 0.8, 0.3 ≤ MPR < 0.8, and MPR < 0.3.
